# Distance supervision as experienced by occupational therapists in mental health: An interpretative phenomenological study

**DOI:** 10.1177/03080226231174102

**Published:** 2023-06-05

**Authors:** Rebecca Matson, Jo Linforth, Chris Edge

**Affiliations:** Department of Occupational Therapy, School of Health Sciences, Institute of Population Health, University of Liverpool, Liverpool, UK

**Keywords:** Supervision, interpretative phenomenological analysis, occupational therapy, mental health

## Abstract

**Introduction:**

Distance methods of supervision rapidly increased in use during the coronavirus disease 2019 (COVID-19) pandemic, and it is important to consider if these methods meet the needs of supervisees when deciding whether to retain these practices moving forward.

**Methods:**

An interpretative phenomenological approach was used to gain insight into the lived experience of distance supervision. Semi-structured interviews were completed with six occupational therapists who had experience receiving distance supervision as a supervisee, all of whom work in inpatient mental health units within a private healthcare company.

**Results:**

Interpretative phenomenological analysis revealed four superordinate themes of an altered interaction; the importance of a protected space; factors within the supervisory relationship and maximising the best of both worlds.

**Conclusion:**

Distance supervision methods afford increased access for supervisees and a reduction in the practical demands when supervised by an off-site supervisor. There are, however, clear differences in the nature of the experience which need to be considered to ensure that supervisee needs are met. This study provides insight into how distance supervision methods impact on the experience for supervisees and suggests areas for further consideration in moving forward with such approaches.

## Introduction

Clinical supervision is widely acknowledged as an important part of practice for occupational therapists, and is intended to both support development of an individual as a health professional and also help to maintain high levels of care within a service (Royal College of Occupational Therapists (RCOT), 2015). A requirement of practice is that an occupational therapist receives, and if appropriate to their job role, provides regular clinical supervision as a means of reflective space that enables continued development throughout their career (RCOT, 2021).

Distance supervision, also known as ‘long-arm’, ‘telesupervision’ or ‘virtual’ supervision refers to supervision via the telephone or a video conferencing platform (Woo et al., 2020). Distance supervision within health care has become increasingly common in recent years, including prior to the coronavirus disease 2019 (COVID-19) pandemic, and is now commonplace within a number of allied health professions (Martin et al., 2017). Yet a rapid evidence review completed by Rothwell et al. (2019) identified that there was insufficient evidence to consider if distance supervision is effective, suggesting that further research is required in relation to this practice.

There are a number of strengths to this format identified in the literature including the ability to access experts within a particular field of practice; improved access to supervision by a member of the same profession and a decrease in the demands of face-to-face supervision such as travel (Martin et al., 2017; Twist et al., 2016). However, there are also added challenges such as decreased nonverbal cues, eye contact, alterations to the natural flow of conversation and decreased sensory feedback (Deane et al., 2015; Miller 2020; Mo & Chan, 2021; Phillips et al., 2021). There is also increased potential for distraction on the part of both the supervisor and supervisee through, perhaps, receipt of emails or ‘zoom fatigue’ (Martin et al., 2016).

There has also been an increase in the demands that the supervisees are facing within their practice, due to the impact of the COVID-19 pandemic, which necessitates a greater focus on staff well-being and support (Martin et al., 2022). An international survey by the World Federation of Occupational Therapists found that during COVID-19 occupational therapists faced increased challenges, including a lack of preparedness, challenges to their own health, decreased morale and anxiety over their own safety and well-being (Hoel et al., 2021). If the supervision space is not effective, there is an increased risk of burnout and impaired decision-making in practice, ultimately leading to lower staff retention, decreased well-being and an impaired service for patients (Kehoe et al., 2021; Martin et al., 2015; Shohet and Shohet 2019; Snowden et al., 2020).

While supervision is acknowledged as high importance within occupational therapy it has received limited attention within the literature in general with only a small number of studies that consider distance supervision (Martin et al., 2015, 2016). The majority of studies have clustered allied health professionals, limiting the capacity to consider factors relevant to the specific profession of occupational therapy. Much focus has been on the experience of students (Boniface et al., 2012; Hunter and Volkert, 2016; Warren et al., 2016) or newly qualified staff with little consideration given to the longer-term experience and benefits of supervision. As the function of supervision can vary depending on the clinical setting (Martin et al., 2016), the focus of this study was narrowed to clinicians working within mental health inpatient settings but considered occupational therapists with varying levels of experience and responsibility. This study seeks to consider the perspective of the supervisee on whether distance supervision is a format that allows their needs to be met and supports them in their practice as an occupational therapist.

The primary aim of this study was to answer the question ‘How is distance supervision experienced by occupational therapists working in inpatient mental health settings?’

The study objectives were:

- To describe the key features of distance supervision as understood by supervisees.
- To explore the perceived effectiveness of this mode of supervision in meeting the needs of supervisees.
- To consider both the barriers and facilitators that may be evident through this medium.
- To increase knowledge on the experience of supervisees receiving distance supervision.

## Method

Interpretative phenomenological analysis (IPA) was identified as a suitable approach to gain an insight into the topic of supervision due to its focus on personal experience (Smith and Osborn, 2007). The experience of supervision is highly subjective; therefore, it is important to consider the experience of each individual before identifying any parity of experience between participants. This would be supported by IPA as it guides the researcher to consider each individual transcript in depth prior to identification of shared themes (Pietkiewicz and Smith, 2014).

Participants were recruited using purposive sampling from within a private healthcare organisation in the United Kingdom, providing a relatively homogenous sample, as is required for IPA (Smith and Nizza, 2022). Participants were in receipt of supervision guided by the same regulations and overarching processes such as company policies and procedures. All participants worked in inpatient mental health settings with the exact nature of the setting varying between adult mental health, child and adolescent mental health and personality disorder services.

Participants were approached via email through a gatekeeper, one of the company’s Occupational Therapy Directors were asked to directly contact the lead researcher (RM) should they wish to participate in the study. They were then provided with a copy of the Participant Information Sheet and written informed consent was obtained through an online consent form.

Semi-structured interviews were conducted via MS Teams and recorded via this platform to allow for verbatim transcription. Consideration was given to face-to-face interviews; however, as well as increasing access to potential participants, it was hoped that completion via this medium may evoke further reflections on distance mediums of interaction. Interviews were completed by the lead researcher (RM) and two other members of the research team (CE and JL). As the lead researcher previously worked in the organisation, any individuals who had received supervision from them were assigned to another member of the team to complete the interview. An interview schedule was developed based on a literature review and the lead researcher’s experience of providing and receiving distance supervision.

### Sample

Six participants took part in the study, a sufficient sample for an IPA study which can have sample sizes as small as one due to the focus on understanding of the subjective experience rather than generalisability (Smith et al., 2009). Data saturation is not the intention of the IPA study, but instead a focus on the considered ‘convergence and divergence’ of experience between participants to provide detailed insight into lived experience (Smith et al., 2009). Demographic information on the participants was not gathered due to the potential that this could make participants identifiable as they were recruited from within one organisation.

All participants had received at least two distance supervision sessions (either phone or virtual) within the last year; were working within inpatient mental health settings and retained a clinical role with a caseload. Occupational therapists in a solely managerial position were excluded due to the potentially significant difference in their role and nature of supervision received.

### Ethical considerations

Ethical approval was obtained from University of Liverpool ILCAMS REC, 2021 and further approval from Cygnet Healthcare Research and Development Committee as the host organisation for the study. Written informed consent was obtained from all participants. Consent was revisited verbally at the beginning of the interview. Participants could withdraw from the study at any point and request to have their data destroyed up until a week following completion of their interview. The option for a debrief was available to all participants.

### Data analysis

Data analysis was completed by the lead researcher (RM) following the stages of IPA analysis (Pietkiewicz and Smith, 2014) which entails analysis of each transcript individually prior to the identification of shared or overarching themes. IPA involves a double hermeneutic, whereby as well as the participants making sense of their experience, the researcher is making sense of the participants doing so (Smith and Nizza, 2022). In the initial stage, early observations and reflections on the language used and the participant’s context and reflexive comments they make during the interview were noted (Smith and Osborn, 2007). Emerging themes were identified from these initial notes to ensure that they retained a strong connection to the participant’s account.

A table of themes was then produced and checked against the participant’s words in the transcript. This process was completed for each transcript with the investigator bracketing ideas from the previous transcript to focus on the individual experience. Once this was completed for each transcript shared concepts between the cases were identified. Themes were then connected with quotes from each participant to demonstrate how each theme applies.

### Trustworthiness

Researcher interpretation forms an important part of IPA analysis and their role as analyst is acknowledged (Smith and Nizza, 2022); therefore, no attempt was made to bracket this. Factors such as the lead researcher’s own experience in delivering and receiving distance supervision while in clinical practice within mental health inpatient settings informed the analysis process. To support the trustworthiness of the findings, an audit trail of each stage of analysis has been retained so that an independent researcher could verify the analysis, and the steps of analysis detailed by Smith et al. (2009) were closely followed. At each stage of the analysis process, themes were checked against participant words to verify their validity.

## Results

Four superordinate themes were identified from the data: an altered interaction; the importance of a protected space; factors within the supervisory relationship and maximising the best of both worlds. Within each of these were sub-themes which will be discussed below. Table 1 provides a thematic overview. To protect confidentiality, pseudonyms are used for all participants.

**Table 1.** Superordinate and sub-themes identified from the findings.

Superordinate theme	Sub-themes
An altered interaction	Impact on openness
	Process as a priority
The importance of a protected space	De-prioritisation of sessions
	External impositions
Factors within the supervisory relationship	Perception of availability
	Establishing a sense of connection
Maximising the best of both worlds	Reducing demands
	Considering the individual

### An altered interaction

All participants discussed the differences in the nature of interactions within distance supervision sessions. These differences are captured by the sub-themes of impact on openness and process as a priority.

### Impact on openness

All participants suggested a difference in openness during distance supervision sessions but with variation between participants as to whether they experienced increased openness when face-to-face or on a video or phone call. Not being face-to-face for sessions could give participants an increased control over what they revealed to their supervisor. Sarah commented:

if something has happened which has actually affected maybe emotional or mental state, sometimes over the phone is, it’s just a little bit easier I think, in terms of, you know like you haven’t got that person seeing you maybe responding a certain way

This led to increased feelings of emotional safety, which was suggested to result in honesty in discussion for some, while for others, they could prevent their supervisor from knowing the full impact of situations on them. Zoe described an ‘expectation of professionalism’ when meeting online, which wasn’t present to the same extent in person. Not being face-to-face also prevented the supervisee from being aware of the full reaction of the supervisor, which for some was a protective factor leading to greater openness. Laura notes:

not having the person there and being able to, read all of their, er like non-verbal cues, has often actually, made, has made things a bit easier for me to talk about, cos I’m not so conscious of, erm, (pause) what they’re communicating

### Process as a priority

Supervision in an online or telephone format automatically became more process driven and focused, losing the personal element. Dan commented:

like now speaking to you via telecommunication, it’s not the same as speaking to you face-to-face and, (pause) so, it is, it feels bit more clinical in that regards

This could support increased focus within the supervision session and for some participants a greater feeling that all areas were being considered. However, supervision lost an element of responsiveness and human interaction, becoming more focused on completion of a form as Jenna described:

when it’s, virtual, you know, you’ve got your, supervision form up and you use that kind of like as an agenda and you’re just kinda filling in the boxes really.

Sarah noted that the session could also feel more restricted by time expectations, seeing the session as agreed for a set time period and no more:

I always feel like it’s, I can fit you in in this time, and then because it comes up on your Outlook, it’s like ok between twelve and one that’s the allocated time I’ve given you, so you’ve only got until one o’clock.

This led to barriers such as feeling unable to impose on a supervisor’s time for any longer than what was allocated, impacting how much would be discussed.

### The importance of a protected space

The majority of participants alluded to the impact of the medium of supervision on the maintenance of a protected space, both physically and in relation to perceived importance of sessions, which is evident in the sub-themes of de-prioritisation of sessions and external impositions.

### De-prioritisation of sessions

When participants discussed online or on phone sessions, they often referred to ‘fitting in’ the session around completing other tasks such as patient care. Supervision decreased in priority and became more focused on efficiency of time, with Jenna describing:

when it’s on Teams, you just kind of, (pause) doing what you need to do and have those discussions and, get it done.

Online supervision was easier to rearrange or cancel due to the decreased investment it required, impacting the perceived sense of priority. Cara stated:

it’s much harder to ignore somebody who has turned up at your door, for supervision. If I was physically there, that’s harder to ignore isn’t it?

There was a perception that face-to-face sessions required greater investment from both parties leading to an increased reluctance to change these or prioritise other obligations.

### External impositions

Supervision also became less protected by and from others, with most participants describing incidents of sessions being interrupted in a way they would not have been if the supervisor were there in person. Dan stated:

I you know, put a sign on the door saying Please Do Not Disturb but people don’t always read signs and can just sort of barge in and if you’re trying to have a really sensitive conversation with someone, and someone opens the door or even they see the sign and they still think ‘no, what I need to say is more important than your privacy’

Supervisees also had increased concern that their supervisor was not fully attentive or more prone to distraction and a temptation to multitask. Cara commented:

sometimes I can hear my supervisor moving around in the background. . .so I think I know you’re at home and you’re making a snack or you’re making, on the phone and I can find that distracting sometimes.

There could also be concern as to the confidentiality of the session and who may overhear the conversation. as described by Sarah:

you don’t know what that other person is doing in the background, or, you know, who’s there in the background either. So I suppose like there’s also that side of things

However, for other participants this was more of a concern when face-to-face within a hospital setting.

### Factors within the supervisory relationship

All participants suggested that the nature of their relationship with their supervisor was a highly significant factor in their experience and could buffer many challenges experienced due to medium of delivery. This relates to the sub-themes of perception of availability and establishing a sense of connection.

### Perception of availability

Overall, participants accepted supervision via distant means when this led to increased scope for contact and when needed, rather than being restricted to a set time. For Dan this was a benefit:

I needed it more sort of like ad hoc in regards to phone calls and emails, that’s where I’ve needed like contact with as. . . my supervisor more, rather than the sort of traditional, you know, four weekly to six weekly supervision

Jenna highlights the benefits of this increased accessibility, making supervision more responsive to individual need and strengthening the relationship as a whole.

I won’t have to think oh right we’ve gotta plan it in and, book it in, we can just say right let’s just have a quick Zoom supervision and get that addressed

Another factor in this was the availability of support from team members, including from other disciplines within their hospital. Zoe noted:

we talk within kind of the MDT around challenges we’re having with patients and I think, I’ve learnt a little more kind of how to use the team around me for those challenges rather than waiting for supervision

While additional support did not negate the need for supervision, it altered the focus of sessions and the supervisee’s response to not having an on-site supervisor.

### Establishing a sense of connection

All participants described a difference in the sense of rapport and connection they experienced but struggled to fully explain this. This was more significant where there had been no prior relationship to virtual sessions. Zoe commented:

it doesn’t come naturally and maybe in time it will, and this may be (laughing) the normal way of building relationships but right now just asking people about kind of personal life or, you know, or ‘how are things?’, you know, ‘what other things do you do outside of work?’, doesn’t really feel appropriate via Zoom

Cara described this as a barrier to the relationship stating, ‘there’s something about being in a room with somebody, erm, that’s different to being on a screen with somebody. There’s a warmth lost’. Most participants felt it was still possible for that sense of relationship to be formed but that depended on the approach of the supervisor, and their ability to convey the same level of support via distant means. Laura suggests:

I think it is partly, or largely even, the majority is due to, (pause) the person that you’re getting support from and how, comfortable you feel with them to see their support and, how willing they are to offer it

There was an apparent need for increased investment in the process from both parties to achieve this dynamic.

### Maximising the best of both worlds

All participants identified some benefits in receiving supervision via a distance medium, but with varying perspectives on how much of the practice should remain. There were two sub-themes within this of reducing demands and considering the individual.

### Reducing demands

Being able to access supervision via video or phone call lessened the practical demands on both parties and also provided added flexibility as to where the session could take place. Dan commented:

you know it starts at one and, you know, you’ve got your cup of tea and your, all your notes, and you’re ready to go at one. And equally when you’ve finished, you know, you sort of finish I don’t have to drive home or, you know, leave or anything like that, so it’s become a bit more efficient

This provided supervisees with a greater sense of control and for some a more positive attitude towards the sessions as this allowed them to more flexibly balance demands on their time, as described by Sarah:

you’re able to kind of dictate where you’re going to be for that supervision, erm. You can even do it where you, you’re, you can be in the car on the phone, erm, or, I’m I’m working from home today so you know I would be able to do it on a working from home day and sort it that way

### Considering the individual

While all participants saw benefits from both face-to-face and distance supervision sessions, there was a clear need to consider individual circumstances and preferences to support a positive supervisory relationship. Jenna expressed how this led to an increased difficulty for her:

I think because I was having challenges anyway, with my own, mental health, I just found that quite, overwhelming. . .I felt they were telling me things that I wasn’t doing, (pause) well, but still not being able to have that, support

Sessions that take place without any face-to-face contact were sufficient for those who were very established in their role and often utilised their sessions for more operational discussions or managerial support, but less so for those who needed a greater sense of support and containment.

There was also a need to consider individual personalities and responses and the impact this may have on the outcome of sessions for that individual. Cara describes ‘I’m not a person who’s tends to send emails, I’m always a person who’ll go and find somebody, to have a discussion, but I think that’s me as a person’. Laura suggested the need for personalisation stating:

yeah if the consistency isn’t’ there, and the approach, isn’t what is right for the person that’s being supervised, then, the experience super, supervision, whatever it would be would, for me personally, erm, would be negatively, erm, affected

A number of participants expressed a preference for a mix of face-to-face and distance supervision sessions interspersed, with others demonstrating a strong preference for one medium highlighting, the importance of this consideration.

## Discussion and implications

This study sought to provide insight into the lived experience of occupational therapists in mental health inpatient settings receiving distance supervision. While previous literature has given limited consideration to the specific experience of occupational therapists, the findings of this study echo themes from research into the experience of other professionals and that of allied health professionals as a collective group. Within this study, the strength of the supervisory relationship appeared to be a more significant a factor than the medium, which is to be expected given findings in supervision literature in general supporting the centrality of the supervision relationship within the process in general (Martin et al., 2022; Rothwell et al., 2021).

Mo and Chan (2021) found that when this relationship was established within a virtual environment, developing a sense of trust is of increased importance and difficulty. A factor within this may be the fact that emotions were more easily concealed via distanced mediums both on the part of the supervisee and supervisor, which had a variable impact on both rapport and the openness of the supervisee. While being able to hide their emotions from their supervisor gave an increased sense of control to supervisees, it is questionable whether being able to conceal the impact of their situation is a good thing, especially during a period such as the COVID-19 pandemic where the restorative function of supervision was found to gain increased significance (Martin et al., 2022).

Supervisees within this study, overall, were more accepting of distance supervision when there was a perceived increase in availability of the supervisor outside of formal supervision sessions and appreciated the increased flexibility offered by these mediums, a finding that was also evident in the study by Mo and Chan (2021). This increased contact led to supervisees feeling more valued and that supervision was more responsive to their needs.

While the theme of the importance of a protected space is less present in the literature in relation to distance supervision this is a theme that is prevalent in relation to supervision in general. Kehoe et al. (2021) found that having a protected time for supervision and what they experienced as a safe, supportive space could significantly influence an individual’s experience of supervision. Added barriers to this within the virtual environment appear to be that there can be a perceived increase in distraction on the part of the supervisor, similar to that identified in a previous study by Martin et al. (2016), and a tendency for a higher level of interruption and rearranging sessions. This also led to concerns of who may be in the background, unknown to the supervisee and what their supervisor may be doing, which has a potentially significant impact on the development of trust, a factor that has been identified as one of the most important within a supervisory relationship (Rothwell et al., 2021).

With the benefits that supervision can have in relation to staff well-being, staff retention, job satisfaction and patient outcomes (Kehoe et al., 2021; Martin et al., 2022; Rothwell et al., 2021), it is vital that ways to make supervision the most effective are considered and good practice is implemented. This is especially significant as we move forward from the pandemic which had led to increased attrition from healthcare professions, added challenges to healthcare staff well-being and mental health and additional barriers to effective patient care (Hoel et al., 2021; Martin et al., 2022).

Training for supervisors and organisational support have been highlighted in the literature as central factors in ensuring the efficacy of supervision (Kehoe et al., 2021; Rothwell et al., 2021), and this need is perhaps even greater when supervision is delivered via video or phone call to support supervisors in developing ways to establish a successful and supportive rapport in an environment with decreased or an absence of nonverbal cues.

### Limitations

The small-scale nature of this study and analysis approach taken mean that the results are not generalisable; however, they do provide insight into the lived experience of distance supervision. Half of the participants recruited provided supervision to others through distance means as well as receiving it via this medium, which at times altered the focus of discussion from their experience as a supervisee, which may have impacted on the depth of insight into the supervisee’s experience. The sample was also from within a private organisation with established structures for supervision; therefore, this would need to be considered when applying the findings outside of this setting.

### Implications

This study provides insights that may be valuable for occupational therapy both in the United Kingdom and beyond due to the increase in virtual practices in many settings following the pandemic. Increased attention is needed on how to ensure that an effective supervision relationship is established when supervision is delivered via distance means to ensure that the needs of each individual are met as much as possible. Consideration is needed of how best to prepare supervisors to deliver supervision via distance means and how to establish a relationship that is conducive to open discussion. Ensuring effective supervision is available may be an important part of reducing levels of attrition and improving staff retention within the profession.

## Conclusion

Distance supervision methods provide ease of access to support, increased flexibility and a reduction in overall demands on both supervisee and supervisor. The ability to receive supervision via such means allowed for continued access during the COVID-19 pandemic when face-to-face sessions were unable to take place for those with off-site supervisors for prolonged periods of time. However, many had to adapt to such mediums with little notice and perhaps insufficient preparedness for the difference in experience this entailed. Further research is needed into ways to ensure that the relational aspects of supervision can be facilitated in a virtual environment and how both supervisees and supervisors can be prepared to adapt in response to these differences.

**Key findings**

- Distance supervision can reduce the demands on supervisees and provide increased accessibility to ad hoc support.
- Choice of medium should be guided by supervisee preference, circumstances and development of rapport.

**What the study has added**

This study has provided insight into the experience of distance methods of supervision and highlights the need for further consideration of how to ensure this meets the needs of occupational therapists.
